# How Does L1 and L2 Exposure Impact L1 Performance in Bilingual Children? Evidence from Polish-English Migrants to the United Kingdom

**DOI:** 10.3389/fpsyg.2017.01444

**Published:** 2017-09-04

**Authors:** Ewa Haman, Zofia Wodniecka, Marta Marecka, Jakub Szewczyk, Marta Białecka-Pikul, Agnieszka Otwinowska, Karolina Mieszkowska, Magdalena Łuniewska, Joanna Kołak, Aneta Miękisz, Agnieszka Kacprzak, Natalia Banasik, Małgorzata Foryś-Nogala

**Affiliations:** ^1^Psycholinguistics Lab, Faculty of Psychology, University of Warsaw Warsaw, Poland; ^2^Psychology of Language and Bilingualism Lab, Institute of Psychology, Jagiellonian University Krakow, Poland; ^3^Early Child Development Psychology Laboratory, Institute of Psychology, Jagiellonian University Krakow, Poland; ^4^Institute of English Studies, University of Warsaw Warsaw, Poland

**Keywords:** bilingual children, L1 acquisition, migrant children, Polish-English bilinguals, home language, minority language, language exposure, language input

## Abstract

Most studies on bilingual language development focus on children’s second language (L2). Here, we investigated first language (L1) development of Polish-English early migrant bilinguals in four domains: vocabulary, grammar, phonological processing, and discourse. We first compared Polish language skills between bilinguals and their Polish non-migrant monolingual peers, and then investigated the influence of the cumulative exposure to L1 and L2 on bilinguals’ performance. We then examined whether high exposure to L1 could possibly minimize the gap between monolinguals and bilinguals. We analyzed data from 233 typically developing children (88 bilingual and 145 monolingual) aged 4;0 to 7;5 (years;months) on six language measures in Polish: receptive vocabulary, productive vocabulary, receptive grammar, productive grammar (sentence repetition), phonological processing (non-word repetition), and discourse abilities (narration). Information about language exposure was obtained via parental questionnaires. For each language task, we analyzed the data from the subsample of bilinguals who had completed all the tasks in question and from monolinguals matched one-on-one to the bilingual group on age, SES (measured by years of mother’s education), gender, non-verbal IQ, and short-term memory. The bilingual children scored lower than monolinguals in all language domains, except discourse. The group differences were more pronounced on the productive tasks (vocabulary, grammar, and phonological processing) and moderate on the receptive tasks (vocabulary and grammar). L1 exposure correlated positively with the vocabulary size and phonological processing. Grammar scores were not related to the levels of L1 exposure, but were predicted by general cognitive abilities. L2 exposure negatively influenced productive grammar in L1, suggesting possible L2 transfer effects on L1 grammatical performance. Children’s narrative skills benefitted from exposure to two languages: both L1 and L2 exposure influenced story structure scores in L1. Importantly, we did not find any evidence (in any of the tasks in which the gap was present) that the performance gap between monolinguals and bilinguals could be fully closed with high amounts of L1 input.

## Introduction

Many studies examining early bilingualism in migrant populations focus on the development of the majority language^1^ (i.e., L2, e.g., Gutiérrez-Clellen et al., 2008; Paradis, 2009; Chondrogianni and Marinis, 2011, 2012; Verhoeven et al., 2011; Hoff et al., 2012). This is because proficiency in the majority language is a prerequisite of success in education (e.g., Strand et al., 2015) and on the job market in the new country (e.g., Shields and Price, 2004; Guven and Islam, 2015). One exception to the predominance of studies on L2 is research on heritage language speakers, conducted mostly in the North American context (e.g., Montrul, 2008; Rothman, 2009; Montrul and Ionin, 2010). A heritage language is understood as “a language spoken at home or otherwise readily available to young children,” but not dominant in the larger society (Rothman, 2009, pp. 156), i.e., it is defined in the same way as we define a minority language in the current paper. While there are many studies on grammatical performance of heritage speakers in L1 (e.g., Polinsky, 2008; Rothman, 2009), there are only a few studies on other aspects of heritage language such as vocabulary and phonology (e.g., Montrul, 2010). Moreover, few of the heritage speaker studies concentrated on the language acquisition process in children (e.g., Montrul, 2008; Polinsky, 2011), but rather on its outcomes in adulthood (for discussion see Rothman, 2009; Rothman and Treffers-Daller, 2014). Overall, although research shows that maintaining the minority language (L1) is of great importance for both well-being of an individual (Portes and Hao, 1998; Yu, 2013; De Houwer, 2015) and for language preservation at the community level (Potowski, 2013), only a few studies have thoroughly examined the development and maintenance of children’s L1 (Rodríguez et al., 1995; Winsler et al., 1999; Gathercole and Thomas, 2009).

We aim to fill this gap by investigating L1 developmental patterns in migrant children raised bilingually. We focus on 4–7 year old Polish-English migrant children living in the United Kingdom. The choice of this particular language group was driven by an unprecedented influx of Poles to the United Kingdom since Poland joined the EU in 2004. The Polish community in the United Kingdom has now reached one million (White, 2011; Kułakowska, 2013), and each year c.a. 25,000 children are born to Polish families (Office for National Statistics [ONS], 2014). This offers an opportunity for systematic and large-scale research on bilingual language development in Polish children, a community that, to our knowledge, has not been thoroughly addressed in the existing research. Although migrant communities of similar sizes exist in other countries, this one seemed especially appropriate for the purpose of studying bilingual language development because of the characteristics of this wave of Polish migration to the United Kingdom. The group, unlike most migrant communities studied so far, does not comprise exclusively unskilled workers with low socio-economic status (SES), which might bias the result. A majority of post-accession migrants from Poland to the United Kingdom were people with secondary education, many of them also holding academic degrees. Also, they were mostly young adults, often bringing young children with them or having children while staying in the United Kingdom (Okólski and Salt, 2014). For this reason, in the current paper, we take a “snapshot” of this new bilingual population and compare the bilinguals’ home language performance to that of their Polish-speaking monolingual peers raised in Poland. We also looked at the age-related differences in the two groups. We aimed to establish to what extent the bilingual migrant children and their monolingual peers in the home country differ in their L1 abilities across four domains of language, i.e., vocabulary, grammar, phonological processing, and discourse. Moreover, our goal was to examine how language experience (in both L1 and L2) influences L1 performance of bilinguals, while controlling the sources of variance related to their general cognitive abilities and socioeconomic status.

### Bilingual vs. Monolingual Language Development

Studies focusing on L2 development in bilinguals demonstrate that bilingual children lag behind their monolingual peers in most aspects of language processing, often scoring similarly to monolinguals with specific language impairment (SLI; Kohnert et al., 2009; Ebert and Kohnert, 2016). Studies investigating L1 in bilinguals offer less conclusive results (e.g., Umbel and Oller, 1994; Winsler et al., 1999), but many indicate a performance gap between bilinguals and their monolingual peers (e.g., Fabiano-Smith and Barlow, 2010). Indeed, research on adult heritage speakers indicates that literacy and formal education in the majority language (L2) often results in the incomplete heritage (L1) language acquisition (Montrul, 2008). As Sorace (2005) points out, this is because the language input heritage speakers receive varies in terms of quality, as heritage speakers are exposed to the input in the minority language mostly from their parents, whose language may have already attrited. However, the differences between monolingual and bilingual children should not conceal similarities between the two developmental paths. Several studies suggest that bilinguals achieve the developmental milestones (defined as the age when the child begins acquiring a particular language skill) roughly at the same time as monolinguals do. This is true for lexical development (Pearson et al., 1993; Hoff et al., 2012), grammatical development (Paradis and Genesee, 1996; De Houwer, 2005; Genesee and Nicoladis, 2007; Paradis, 2009) and phonological development (Fabiano-Smith and Barlow, 2010). For example, both bilinguals and monolinguals utter their first words around the age of one, and have similarly sized vocabulary and phonological inventory, when both languages of the bilingual are taken into consideration (Fabiano-Smith and Barlow, 2010; Hoff et al., 2012). There is also evidence that the abilities to produce coherent discourse do not differ between bilinguals and monolinguals of a comparable age (e.g., Paradis and Kirova, 2014). In other words, there are both similarities and differences between monolingual and bilingual developmental paths. Research findings suggest that the bilingual development has its own specificity, and that monolingual norms should not be applied to bilingual speakers (Gathercole, 2013a,b; Armon-Lotem et al., 2015).

In the subsequent sections, we briefly review the literature related to the bilingual development in the four language domains that are the focus of our study: vocabulary, grammar, phonology, and discourse. For each language domain, we address two critical issues: the differences between bilingual children and their monolingual peers, and the impact of language exposure on performance in each of the language domains in L1 and L2.

#### Vocabulary

Studies examining L2 vocabulary in bilingual children consistently report that bilinguals lag behind their monolingual peers on both receptive tasks (Bialystok et al., 2010; Verhoeven et al., 2011) and productive tasks (Uccelli and Páez, 2007). Some studies even find typically developing bilingual children to have smaller receptive vocabularies in L2 than monolinguals with SLI (Verhoeven et al., 2011).

In terms of L1 vocabulary size, some studies suggest that bilingual children raised in the migrant setting are disadvantaged (e.g., Pearson et al., 1997; Uccelli and Páez, 2007). Other studies indicate that L1 vocabulary in bilinguals is not affected negatively, either in the receptive tasks (Umbel and Oller, 1994; Winsler et al., 1999; Leseman, 2010), or in the productive tasks (Leseman, 2010). Thus, the results are inconclusive and they should be treated with caution, since the majority of L1 vocabulary studies compared children’s lexical acquisition between the two languages of bilinguals, and did not compare bilinguals’ L1 scores to the vocabulary scores of a matched monolingual group.

The observed discrepancy in the results on L1 vocabulary in bilinguals may stem from methodological issues (e.g., the lack of well-matched control groups), but also from the variability in exposure to languages. Previous research indicates that L1 vocabulary size is closely connected to the reported amount of L1 exposure, while L2 vocabulary size is related to exposure to L2 (Pearson et al., 1997; Vermeer, 2001; Patterson, 2002; De Houwer, 2007; Thordardottir, 2011; Hoff et al., 2012; Hoff and Core, 2013). This relationship is especially strong for the productive vocabulary. For example, in a study on English-French bilingual children in Canada, the participants with equal amounts of exposure to L1 and L2 had native-like scores in a receptive vocabulary task, but not in a productive vocabulary task. To perform on par with the monolinguals in the productive vocabulary task, the children needed to have more exposure in the language tested (Thordardottir, 2011). Moreover, Pearson et al. (1997) established the 20% threshold hypothesis – they claim that children who hear less than 20% of their input in a given language are often unwilling to speak that language. In line with this hypothesis, Hoff et al. (2012) suggests that 20% is an absolute minimum of input for a child to be able to use a language. Studies on heritage speakers also suggest that vocabulary in L1 is affected by both the amount and quality of input in L1 (Schwartz, 2008).

Overall, the current literature indicates that bilingual children have significantly lower vocabulary scores in L2, compared to their monolingual peers, while the findings regarding L1 vocabulary are inconclusive. In general, the amount of exposure seems to be crucially linked to vocabulary performance of the bilingual children, especially in language production.

#### Grammar

The studies examining specific areas of grammar in bilingual development show mixed results. On the one hand, some reported that bilinguals acquire certain structures in L2 (e.g., such as finite verb forms, Paradis and Genesee, 1996) just like their monolingual peers, especially when L2 is their dominant language (see De Houwer, 2005; Conboy and Thal, 2006; Genesee and Nicoladis, 2007; Parra et al., 2011). Still, many studies suggested that bilingual children perform worse than monolinguals on L2 grammar tasks, for example the ones examining the application of tense morphology (see Hoff et al., 2012). The bilingual disadvantage seems to be smaller for the receptive than productive tasks (Verhoeven et al., 2011; Chondrogianni and Marinis, 2012). Moreover, the majority of grammatical errors reported in studies on bilingual acquisition appear to be developmental errors (for review see Paradis, 2009). As for global L2 grammar measures, including the Sentence Repetition task (SRep; see Marinis and Armon-Lotem, 2015), which involves verbatim repetitions of sentences with various grammatical structures in the target language, bilingual children usually score lower than monolinguals (Verhoeven et al., 2011; Komeili and Marshall, 2013; Thordardottir and Brandeker, 2013). When it comes to grammatical systems of L1 in the minority speakers, they are often simplified as regards the development of certain grammatical structures (see Benmamoun et al., 2013; Scontras et al., 2015). Bilingual children can also score lower on L1 holistic grammatical assessment tasks such as the SRep, especially if they did not have much exposure to that language (Thordardottir and Brandeker, 2013). The areas of L1 grammar that appear to be particularly problematic include agreement morphology (e.g., Bolonyai, 2007; Montrul and Potowski, 2007; Polinsky, 2008; Gathercole and Thomas, 2009), overusing rigid word order patterns (e.g., Isurin and Ivanova-Sullivan, 2008), or applying and interpreting long-distance binding (e.g., Polinsky, 2006; Kim et al., 2009). However, since many of these accounts come from studies on older participants than preschool children, it is necessary to further investigate at which point in development those alternations in syntax emerge (Polinsky, 2016). In a study focused specifically on child minority language, Montrul and Potowski (2007) investigated the acquisition of Spanish gender agreement in school-aged heritage speakers of Spanish enrolled in a dual Spanish-English immersion program. As evidenced by the data coming from an oral narrative task and a picture matching task, the heritage speakers scored lower than Spanish monolinguals but higher than the L2 learners in applying gender agreement rules to determiners and adjectives.

Overall, research indicates that poorer performance on L1 grammatical tasks might be related to impoverished or altered exposure to L1 or to the influence of the dominant community language (see Rothman, 2007; Gathercole and Thomas, 2009; Benmamoun et al., 2013; Scontras et al., 2015; Hoff et al., 2017). For example, in Spanish-English bilingual children, L1 exposure at home has been found to be related to scores in L1 (Spanish) grammaticality judgment task targeting the knowledge of gender marking and *that*-trace structures (Gathercole, 2002a,b). In Welsh-English bilinguals, home and school exposure to the L1 minority language (Welsh) correlated with children’s receptive command of the syntactic patterns of Welsh gender marking and the use of word order cues in identifying subjects (Gathercole and Thomas, 2009). Montrul and Potowski (2007) observed that sequential bilinguals, who were first exposed exclusively to Spanish as an L1, and thus received more overall exposure in that language, outperformed simultaneous bilinguals in applying gender agreement rules to adjectives. The results showed that the development of certain aspects of L2 grammar may be affected by reduced exposure in early childhood.

There is also evidence suggesting that structures from the dominant language might be incorporated into the weaker language more often than the other way around (Döpke, 1998; Yip and Matthews, 2000). For instance, the effects of L2 exposure on the L1 minority language may affect some specific areas of L1 grammar, such as the use of overt versus null subjects (e.g., Paradis and Navarro, 2003), determiners (e.g. Kupisch, 2007; Montrul and Ionin, 2010) or inflectional morphology (see Benmamoun et al., 2013). However, it is often hard to disentangle the effects of L2 transfer from the effects of the reduced input in L1 (Scontras et al., 2015).

Overall, many studies suggest that bilingual children may experience developmental difficulties in the domain of morphosyntax in their non-dominant language, whether L1 or L2. Crucially, however, the gap between the performance of mono- and bilingual groups has been found to depend on the amount and type of exposure to the target language.

#### Phonology

Bilingual children can differ from their monolingual peers in terms of phonological development in L1 and L2 in three ways: delay, acceleration, and transfer. First, bilinguals might learn to produce some speech patterns (e.g., vowels, Kehoe, 2002; consonants, Goldstein and Washington, 2001; prosody, Lleó, 2002) later than monolinguals. Moreover, when tested in L2 on generalized phonological assessment measures such as English Diagnostic Evaluation of Articulation and Phonology (Dodd et al., 2002), bilingual children might obtain low scores, which in monolinguals would be typical for phonological delay (En et al., 2014). The delay in the acquisition of phonological features of L1 has also been reported (Goldstein and Washington, 2001), but not in all studies (Kehoe, 2002).

Secondly, bilinguals might acquire some phonological features in L2 faster than their monolingual peers. For instance, Polish-English bilinguals and Welsh-English bilinguals acquire complex consonantal clusters in English faster than their monolingual peers, most likely due to the fact that their L1 is rich in complex consonant clusters (Mayr et al., 2014; Tamburelli et al., 2015). To our knowledge, there have been no studies showing a similar effect for L1 in bilingual speech.

Thirdly, bilinguals might exhibit phonological transfer, i.e., pronounce the sounds in one language with the phonetic features of their other language. Phonological transfer between bilinguals’ two languages may affect both prosodic patterns (Paradis, 2001) and segmental features (Fabiano-Smith and Barlow, 2010; Barlow, 2014) and can take both directions, i.e., from L1 to L2 and from L2 to L1 (Fabiano-Smith and Barlow, 2010; Fabiano-Smith and Goldstein, 2010; Marecka et al., 2016). Overall, while bilingual children do not have smaller phonological inventories than monolinguals, they tend to mix the phonological features of both languages (Fabiano-Smith and Barlow, 2010). Heritage language studies suggest that these tendencies might carry into adulthood of the bilingual speakers. L1 phonological features in the speech of adult heritage speakers such as vowel quality or VOT can shift toward L2-like values (Godson, 2004; Nagy and Kochetov, 2013), even though the L1 accent of these adult heritage speakers is reported to be more native-like than the accent of L2 learners of a particular language (Au et al., 2002; Oh et al., 2003).

Apart from testing for the ability to produce appropriate phonemes in the target language, several studies used the non-word repetition (NWR) task to study phonological processing in bilingual children. When the non-words used in the test are highly L1- or L2-like, they tend to measure the inventory of phonological representations of a child (Jones et al., 2010; Jones, 2011). Bilinguals perform worse than monolinguals on the NWR with L2-like non-words (Kohnert et al., 2006), sometimes even on par with monolinguals with SLI (Windsor et al., 2010). When tested in their L1 (and not L2) bilinguals tend to perform better (Gutiérrez-Clellen and Simon-Cereijido, 2010; Summers et al., 2010). When non-words are quasi language-universal, bilinguals perform similarly to their monolingual peers (Boerma et al., 2015).

Both phonological development and processing are influenced by the cumulative language exposure. Many studies of phonological development have reported that children who started acquiring L2 earlier (i.e., cumulatively had more exposure to L2) sound more native-like than children who started acquiring the language later (Asher and García, 1969; Snow and Hoefnagel-Höhle, 1977; Flege and Fletcher, 1992; Flege, 1995; Aoyama et al., 2008). Moreover, the phonological performance in both L2 and L1 is directly proportional to the exposure and use of a particular language (Flege, 2002). Phonological processing (as measured with NWR) is also connected to the amount of exposure that bilinguals receive in the tested language (Summers et al., 2010), although to a smaller degree than vocabulary (Thordardottir and Brandeker, 2013).

#### Discourse

In studies of discursive abilities, children are usually asked to narrate a story, often based on pictorial stimuli. Narrative data support the results from standardized tests by providing additional performance measures across the languages of the bilingual child (Iluz-Cohen and Walters, 2012). A measure usually taken into consideration here is the structural coherence of narratives, i.e., the story structure, which is subsequently assessed in terms of how well the child refers to the goals of the characters, the attempts to reach these goals and their outcomes (Gagarina et al., 2016; see also Stein and Glenn, 1979). Story structure scores go beyond the assessment of single words or sentences, but instead indicate the level of more complex cognitive and pragmatic abilities (Gagarina, 2016). Studies comparing the story structure of bilinguals in L2 or L1 with that of their monolingual peers are infrequent and their results are mixed. One study comparing L1 structural coherence in bilingual Finnish-Swedish children with that of Finnish monolinguals found no differences between the two groups of children (Kunnari et al., 2016). On the other hand, in a study comparing the performance in L1 Russian of Russian-Norwegian children to Russian monolinguals, the bilinguals scored lower on the story structure in their L1 (Rodina, 2016). The same pattern has also been observed in the studies on heritage speakers. In a case study by Polinsky (2008), two heritage speakers of Russian (a 9-year-old and a college student) were found to produce significantly shorter utterances and narrate at a slower pace than monolingual Russian speakers.

The effect of language exposure on children’s narrative abilities is a complex issue. On the one hand, some findings suggest that the exposure to a particular language might not be crucial to narrating in that language. Most studies comparing bilingual children’s narrative abilities in L1 and L2 indicate that the structure of narratives is relatively invariant across languages and that the measures of the story coherence in the child’s two languages tend to be highly correlated (Muñoz et al., 2003; Fiestas and Peña, 2004; Uccelli and Páez, 2007; Gagarina, 2016; Kunnari et al., 2016). In general, children produce equally coherent stories in both languages, even if the child’s linguistic abilities in terms of vocabulary or grammar in one of the languages are weaker (Gagarina, 2016). The finding that the story structure does not differ across the languages of a bilingual is probably related to the fact that the ability to tell coherent stories taps into the child’s general knowledge about the world and thus seems to be relatively language-independent (Gagarina, 2016; Gagarina et al., 2016). This would indicate that language-specific exposure might not be crucial for developing narrative skills.

On the other hand, several studies point to the importance of language exposure, showing that the narrative structure in bilinguals might be better in L1 than in L2 (Kapalková et al., 2016; Roch et al., 2016). A study on L1 Russian narratives in Russian-Norwegian preschoolers suggests that the L1 story structure might be dependent on the amount of exposure to L1 (e.g., Rodina, 2016). Further, as indicated by Gagarina (2016), the strong positive correlations between the story structure in L1 and L2 cease to occur after several years of schooling in the majority language. Then, the stories told in the language of schooling become more coherent than those in the home language. This result suggests that the story structure, rather invariant across languages in young bilinguals, might be sensitive to explicit narrative teaching at school and to receiving large amounts of structured input and modeling in the majority language. Finally, several studies showed that older bilingual children produce more coherent stories than younger children (Bohnacker, 2016; Maviş et al., 2016). This might be attributable to children’s cognitive maturity, but also to the differences in language exposure.

To conclude, bilingual children’s discursive abilities are rather under-researched in comparison with other aspects of language use, and the results of studies are not clear-cut. Some suggest that the narrative abilities of bilinguals might be influenced by exposure and modeling, especially at the later stages of education. However, the results of studies on the narrative abilities in bilingual preschool children suggest that producing coherent stories is an area where bilinguals and monolinguals might perform similarly, regardless of the L1 exposure.

### The Current Study

The literature review presented above reveals a rich body of research devoted to language acquisition in bilingual children. However, it is clear that despite the wealth of studies, many facets of bilingual language acquisition are still under-researched. The majority of studies focused on the L2 of bilinguals and only few examined their L1 and benchmarked it against a monolingual control group (e.g., Umbel and Oller, 1994; Thordardottir, 2011; Thordardottir and Brandeker, 2013). Moreover, only few studies investigated several different language measures on the same group of participants (Uccelli and Páez, 2007; Verhoeven et al., 2011; Thordardottir and Brandeker, 2013). Thus, there is certainly a need for large-scale investigations that would allow to obtain a comprehensive picture of differences in the linguistic performance between monolinguals and bilinguals by comparing them in different areas of language use. Also, a certain limitation of many previous studies is that they seldom controlled for language exposure in the bilingual group, despite the fact that this single variable can potentially explain many differences between monolinguals and bilinguals (Pearson et al., 1997; Thordardottir, 2011; Thordardottir and Brandeker, 2013). Finally, to our best knowledge, there are no studies which would examine the effect of language exposure on different language domains in child bilingual speakers, while controlling for potentially confounding variables such as short-term memory (STM) capacity, non-verbal IQ, or SES. Controlling these variables seems important, since research consistently indicates their crucial role in language development. STM capacity has been linked to the development of vocabulary (Gathercole et al., 1992) and both vocabulary and grammar (Verhagen and Leseman, 2016) in preschool children. Moreover, deficits in non-verbal IQ might be linked to language deficits (Botting, 2005) and SES might determine the overall language development (see Hoff, 2006 for a review; Hoff, 2013).

#### Measuring Language Exposure

Although it is generally agreed that language exposure plays an important role in language acquisition, the construct is a matter of much controversy (Carroll, 2017). The term “language exposure” lacks an accurate definition and is measured in various ways (see Armon-Lotem, 2016; Carroll, 2017 for discussion). In the present paper, we are following Carroll (2017) and we define exposure as an observable and measurable contact with a particular language.

The quantification of language exposure has been a challenging task. To estimate exposure several related factors can be used: the intensity of contact with a given language (also as a function of the number of interlocutors available for a given language), the age of the first contact with the language, and the time spent while exposed to a particular language. Indirectly, also chronological age might be a contributing factor, because older children tend to have greater length of exposure to a given language in their lifetime. Ideally, all these factors should be disentangled and their contribution measured independently. However, because these predictors are highly correlated, doing so would require testing huge participant samples, and to the best of our knowledge, no study has accomplished this so far. The existing studies that controlled for one of these factors conceded that the other ones were left uncontrolled (e.g., Bedore et al., 2016). One way of solving this problem is to eliminate at least one factor, for example the Age of Acquisition, by testing populations that are exposed to both languages from birth (e.g., testing English-French in bilingual families in Montreal; Thordardottir, 2017). But even then, the contribution of the three other highly correlated variables remains to be controlled. A better way of addressing the problem is to circumvent it by creating one cumulative index that encompasses all the related factors. Such an approach was taken in a few recent studies (Unsworth, 2013; Unsworth et al., 2014; Vender et al., 2016) and it is also chosen in the present study. Such an index typically reflects the length of exposure to a language (from the age of the first contact to the time of testing), obtained from parental questionnaires. Specific approaches to exposure may differ in how exactly this information is elicited via background questionnaires. For example, Unsworth (2013) estimates the percentage of waking hours during which children were exposed to a particular language, in each year of their life. In the present study, we estimated the intensity of contact with Polish and English. We multiplied this estimation by the time before and after migration, respectively. The estimate of intensity of contact was based on the number of speakers at home when the language was used. Hence, our index of language exposure simultaneously reflects both the quantity and quality of exposure (i.e., the number of different speakers). In the methods section, we describe how our index of cumulative language exposure was constructed in more detail.

#### Research Questions

Here, we present a comprehensive analysis of L1 performance in bilingual migrant children, as compared with their monolingual peers, with a number of factors controlled. We used six direct language measures to test over 200 typically developing children (including more than 80 bilinguals) aged 4;0 to 7;5. The measures included receptive and productive vocabulary, receptive and productive grammar (SRep), phonological processing (NWR), and narrative skills. What is more, in the current analyses, we assess the impact of exposure to both L1 and L2 on bilinguals’ performance in each of the language domains.

Our analyses focused on the three main research questions:

(1) What are the differences between bilingual migrant children and their monolingual peers in the four domains of Polish L1 development?
(2) How does the cumulative exposure to L1 and the cumulative exposure to L2 influence performance of the bilingual children in each of the language domains?
(3) Can high exposure to L1 minimize the potential gap between monolinguals and bilinguals?

## Materials and Methods

### Participants

Overall, 173 bilingual children and 311 monolingual participants took part in the study. However, the analyses presented in the current paper were based on subsamples from both groups. In the analyses, we considered only those participants for whom we had a full data set necessary to control for the non-verbal intelligence (*Raven’s Colored Progressive Matrices*; Raven, 2003; Jaworowska and Szustrowa, 2003), STM (forward digit span, Wechsler, 1974), and SES (background questionnaires). We excluded the children who had hearing problems (6 bilinguals, 3.5% of the bilingual sample; 9 monolinguals; 2.9% of the monolingual sample). Additionally, from the bilingual group we excluded the children who were effectively trilingual (15 children; 8.7% of the bilingual sample; see also Mieszkowska et al., 2017), from the monolingual group those who occurred to be bilingual (living in Poland, 3 children, 1% of the monolingual sample) and those at risk of SLI, as indicated by parental concerns reported in the questionnaires (4 bilinguals; 2.3% of the bilingual sample, 3 monolinguals, 1% of the monolingual sample). Eventually, data from 233 children (88 bilingual and 145 monolingual) were considered for further analyses. Seventy of the bilingual children who took part in the study had both Polish-speaking parents. Eighteen children lived in families with a Polish-speaking mother and a father speaking English at home (11 native English speakers and 7 non-native English speakers). All the bilinguals lived in the United Kingdom, but they varied in terms of the age of their first contact with English (*M* = 13 months, *SD* = 16 months). Fifty-five of them were first exposed to English within the first year of life (36 just after birth). Others had their first contact with English later (up to 60th month of life).

For each of the language measures reported in this paper, we conducted separate analyses on a subsample of children. The subsamples consisted of all bilingual children for whom we had the data on the task of interest and a group of monolinguals matched one-to-one to the bilingual group on age, SES (years of mother’s education), gender, non-verbal IQ (Raven scores), and STM (as measured by forward digit span). The matching procedure served to ensure that any differences between the groups can be attributed to language status (bilingual or monolingual), and not to other factors known to affect the performance in the tasks of interest, such as environmental differences related to SES (see Hoff, 2006; Qi et al., 2006; Hoff and Core, 2013), or children’s cognitive abilities (see Kail, 2000). The characteristics of the overall sample and the task-specific subsamples are presented in **Table 1**.

**Table 1:** Demographic information and descriptive statistics for background measures in the participant subsamples.

	In total		Subsample 1 (Receptive vocabulary)		Subsample 2 (Productive vocabulary)		Subsample 3 (Receptive grammar)		Subsample 4 (Productive grammar)		Subsample 5 (Phonological processing)		Subsample 6 (Discourse)	
**Bilinguals**	***N* = 88 (51 F)**		***N* = 87 (50 F)**		***N* = 87 (50 F)**		***N* = 74 (42 F)**		***N* = 80 (46 F)**		***N* = 79 (47 F)**		***N* = 53 (32 F)**	
	***Mean***	***SD***	***Mean***	***SD***	***Mean***	***SD***	***Mean***	***SD***	***Mean***	***SD***	***Mean***	***SD***	***Mean***	***SD***

Age (years; range: 4.36–7.01)	5.69	0.76	5.69	0.76	5.69	0.76	5.76	0.74	5.77	0.73	5.76	0.75	5.71	0.71
Years of mother’s education (range: 10–24)	16.27	2.98	16.32	2.96	16.32	2.96	16.30	3.15	16.45	2.93	16.30	2.89	16.40	2.81
Raven (range: 12–34)	21.91	5.83	21.89	5.86	21.89	5.86	21.86	5.63	22.31	5.83	22.25	5.73	22.02	5.10
Digit span (range: 0–6)	3.90	1.12	3.91	1.13	3.91	1.13	3.88	1.15	3.98	1.04	4.01	0.97	4.08	0.83
Age of first L2 contact (years; range: 0–5.0)	1.08	1.34	1.10	1.34	1.10	1.34	1.17	1.40	1.17	1.37	1.16	1.38	1.09	1.32
L1 (Polish) cumulative exposure (range: 70.83–515.86)	316.45	93.64	316.39	94.19	316.39	94.19	319.93	99.05	326.11	89.54	323.68	91.91	317.37	84.66
L2 (English) cumulative exposure (range: 16.87–362.13)	158.85	81.34	158.36	81.68	158.36	81.68	158.74	86.11	155.11	82.35	157.44	82.39	155.88	77.68

**Monolinguals**	***N* = 145 (74 F)**		***N* = 87 (50 F)**		***N* = 87 (50 F)**		***N* = 74 (42 F)**		***N* = 80 (45 F)**		***N* = 79 (47 F)**		***N* = 53 (31 F)**	
	***Mean***	***SD***	***Mean***	***SD***	***Mean***	***SD***	***Mean***	***SD***	***Mean***	***SD***	***Mean***	***SD***	***Mean***	***SD***

Age (years; range: 3.52–7.23)	5.60	0.72	5.64	0.70	5.63	0.69	5.67	0.74	5.60	0.69	5.70	0.63	5.73	0.66
Years of mother’s education (range: 11.5–25)	17.46	2.69	16.74	2.43	16.80	2.33	16.66	2.58	16.81	2.77	16.88	2.44	16.91	2.96
Raven (range: 11–32)	22.05	5.00	21.90	4.69	21.91	4.84	22.36	4.60	22.23	4.29	22.30	4.63	22.40	5.39
Digit span (range: 0–7)	3.95	0.93	3.97	0.99	3.98	1.02	4.00	1.01	4.01	0.92	4.08	0.94	4.15	0.97

### Materials and Procedures

#### Tasks

The testing battery included six published normed tests or their non-normed adaptations, six experimental tasks used in previous research, six language tasks designed as a part of the Bi-SLI-Poland project within the European COST Action IS0804, and three experimental tasks designed for the project. Below all the tasks are recounted and the tasks used in the current analysis which do not have standardized administration procedures described in the tests manuals are presented in more detail.

##### Receptive vocabulary (Obrazkowy Test Słownikowy – Rozumienie, OTSR)

Children’s receptive vocabulary was measured with *Obrazkowy Test Słownikowy*, OTSR (The Picture Vocabulary Test – Comprehension; Haman and Fronczyk, 2012). Each child was tested with two available versions of the test (A and B) to allow more data points in the assessment. The two versions of the test are fully comparable with each other and are used independently when testing for diagnostic purposes or when a retest is needed in a short period of time. Each version includes 88 items that are ordered from the least to the most difficult. The OTSR assesses the comprehension of nouns, verbs, and adjectives. Each test item is accompanied by four colored pictures. One picture depicts the target word and the three other pictures are foils, which consistently include one phonetic foil, one semantic foil, and one thematic foil.

The child is presented with one word at a time and has to point to one picture out of four that appropriately depicts the word. The child does both versions of the test, with the order of the versions counterbalanced. Depending on the child’s age, the easier, initial items are skipped in each version. The procedure in each version is terminated after four consecutive errors.

Overall, a participant can receive a maximum of 88 points in each version – one point for each correct answer. For the purpose of this study, we considered only one of the test versions, for which a child obtained a higher score. We assumed that this score was more immune to the problems connected with test delivery, such as the child’s boredom, or lack of concentration that led to the early termination of the test.

##### Productive vocabulary (Zadanie Nazywania Obrazków, ZNO)

The productive vocabulary was measured with *Zadanie Nazywania Obrazków*, ZNO (Picture Naming Task; Haman et al., 2012; Haman and Smoczyńska, 2010, unpublished). The task consists of 53 color pictures depicting 32 nouns and 21 verbs presented in the order of ascending difficulty. Each child is presented with all 53 pictures one by one, and is asked to name each picture with one word. The task has to be administered to the last item, regardless of the number of errors made by the child. The child scores a point for each correct answer, which includes the target word, its close synonym, or a dialectal variant. The maximal number of points is 53.

##### Receptive grammar (TROG-2)

We used the Test for the Reception of Grammar – TROG-2 (Bishop, 2003; the Polish translation by Smoczyńska, 2008, unpublished) as a measure of receptive grammar. TROG-2 tests the comprehension of 20 syntactic constructs, organized in blocks A–T with progressing order of difficulty, as established for the English version. Each grammatical construct is included in four test items. The structures tested by TROG-2 include, for example: negatives, singular and plural inflection, object and subject relative clauses, etc. (for the exhaustive list of TROG-2 structure blocks, see Bishop, 2003).

Each test item is presented in a multiple-choice format with four pictures presented on a single board. One of the pictures illustrates the target structure and three constitute the lexical and grammatical foils to this structure. The child is auditorily presented with the stimulus containing a particular grammatical structure. Then the experimenter asks the child to point to one of four pictures which best corresponds to what he/she has heard. For each correct answer the child scores one point, and the maximum number of points is 80. In the Polish version of TROG-2 all children were expected to complete the entire task.

#### Productive Grammar (Sentence Repetition, LITMUS-SRep)

Productive grammar was examined with the Polish adaptation of Sentence Repetition task, LITMUS-SRep (henceforth: SRep, Banasik, Haman, and Smoczyńska, 2012, unpublished), based on the English task SASIT (Marinis et al., 2010). The adaptation is composed of 68 Polish sentences, with varying levels of grammatical complexity. The sentences contain a wide range of grammatical constructions, including negations, questions, passives, object and subject relative clauses, conditionals, object and subject clefts and noun complement clauses. The sentences are morphologically varied and controlled for length (between 5 and 9 words, no more than two clauses) and the properties of the content words used (lexical frequency, age of acquisition). All the sentences were recorded by two native speakers of Polish (male and female).

During task administration, children are asked to listen to the recorded sentences one by one and repeat them as accurately as possible. Each sentence is heard only once. The child is praised for repeating the sentences irrespective of accuracy, but no corrective feedback is given. The repetitions are recorded and then transcribed. The final score reflects the percent of correctly repeated words, relative to all the words in a given sentence (range 0–100).

##### Phonological processing (Non-word Repetition, NWR)

We tested phonological processing with the Polish NWR task, NWR (Szewczyk and Wodniecka, 2012), consisting of 50 non-words. All non-words, recorded by a female native speaker of Polish, are between 2 and 4 syllables long, have a fixed stress pattern on the penultimate syllable (which is the default stress pattern in the Polish language) and are phonotactically legal. Most of the items are highly Polish-like, i.e., they contain consonant clusters and affixes typical for Polish morphology. Sometimes, they also contain lexical morphemes. The recordings of non-words are presented in the order of increasing difficulty. Participants listen to the recordings via headphones and repeat them. Subsequently, the recorded repetitions are transcribed by two independent judges. Based on their transcriptions, each non-word is categorized as either correct or incorrect. Developmental errors are disregarded and treated as correct productions. For each correctly repeated word the child receives one point. The maximal number of points for this task is 50.

##### Discourse (LITMUS-Multilingual Assessment Instrument for Narratives, LITMUS-MAIN)

To assess children’s discursive abilities we used the Polish adaptation of the LITMUS-Multilingual Assessment Instrument for Narratives, LITMUS-MAIN (henceforth: MAIN; Gagarina et al., 2012) by Kiebzak-Mandera et al. (2012). The MAIN consists of four parallel cross-culturally neutral picture stories, each comprising six pictures. Each story includes three episodes (two pictures per episode). The episodes can be described in terms of the GAO sequences: a Goal (i.e., the protagonist wanting something), an Attempt to reach this goal, and the Outcome (e.g., The cat wants to catch a butterfly – Goal; The cat jumps forward – Attempt; The cat falls into the bushes – Outcome). The testing procedure involved two modes, the Telling mode and the Retelling mode.

Each session starts with a warm-up conversation, followed by the Telling mode and the Retelling mode. In the Telling mode, the experimenter presents the child with three envelopes, containing the same picture story. The child is asked to choose one envelope, look at the pictures and tell a story based on the pictures without showing them to the experimenter (the non-shared attention paradigm). In the Retelling mode, the experimenter shows the child another picture story, tells the story to the child and asks the child to retell the story based on the pictures and the model story he/she has heard (the shared attention paradigm). The whole session is recorded and transcribed.

In this study, we assessed the story structure of each narrative (told and retold) in accordance with the MAIN (see Gagarina et al., 2012). The child could get the maximum of 2 points for the setting of the story and then 5 points for each episode including the GAO sequences (1 point for conveying the initial mental state of the character, 1 point for expressing the Goal, 1 point for the Attempt, 1 point for the Outcome, and 1 point for describing the character’s reaction to the outcome), which gives the maximum of 17 points per story.

#### Procedure

All children were tested individually in a quiet room: the monolingual Polish children in their preschools or in their homes in Poland, the bilingual children in their schools or in their homes in the United Kingdom. Apart from the language tasks in Polish described above, each bilingual child was tested with a set of analogous language tasks in English, but these tasks are beyond the focus of the present report. Moreover, all children were tested with a battery of cognitive tasks, including the Digit Span (Wechsler, 1974)^2^ and Raven’s Colored Matrices (Jaworowska and Szustrowa, 2003). The bilingual children were tested on the cognitive tasks only in their dominant language, as declared by their parents. In the case of children whose parents declared that they could not indicate which language was dominant, it was assumed that the child was balanced in their knowledge of the two languages and the language in which the cognitive tasks were performed was randomly selected.

Each monolingual child was tested throughout 3–4 sessions and each bilingual child – throughout 5–7 testing sessions (2–3 sessions in the non-dominant language and 3–4 sessions in the dominant language). Each session lasted approximately 45–90 min including breaks between the tasks. The duration of the session depended on the child’s pace of doing the tasks. The order of the tasks in the testing sessions was counterbalanced across participants. The tasks in Polish were administered by a native speaker of Polish, while the tasks in English (not included in the present report) were administered by a native speaker or a highly proficient user of English. Polish and English were never tested on the same day.

### Calculating the Index of Cumulative Exposure to L1 and L2

In order to statistically control for the language exposure of bilingual children, we calculated an index of cumulative language exposure in L1 and L2. First, we estimated to what extent a child was exposed to each language when living in the United Kingdom on the basis of the Questionnaire for Parents of Bilingual Children [PABIQ – Tuller, 2015; Polish adaptation by Kuś, Otwinowska, Banasik, and Kiebzak-Mandera (2012, unpublished)]. In the questionnaire, we asked parents to estimate on a 5 point Likert scale how often the child was addressed in English and Polish in particular communicative situations such as parents talking to the child, other children talking to the child in the day-care, etc. (0 – not at all, 4 – exclusively in this language)^3^. These scores were aggregated to obtain an estimate of the bilingual children’s exposure to Polish and to English during their stay in the United Kingdom. The maximal score for each language was 91, the actual values for L1 (Polish) were in the 15–67 range (*M* = 45.93, *SD* = 11.63), and for L2 (English) in the 15–61 range (*M* = 36.01, *SD* = 11.31). Because some of bilingual children (16 participants) in our group were born in Poland and only later immigrated to the United Kingdom, we assumed that when living in Poland the children had the maximal exposure to Polish (i.e., 91) and none to English. After immigrating to the United Kingdom, some children regularly spent a considerable amount of time in Poland (e.g., 3 months of summer holidays each year). Thus, we assumed that also during these periods of time the children had the maximal exposure to Polish and no exposure to English.

**FIGURE 1:**
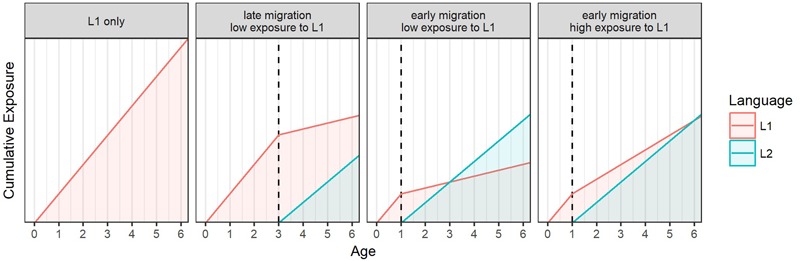
Four examples of how cumulative exposure to L1 and to L2 may change with age. The intensity of exposure corresponds to the line slopes, whereas the position on the Y-axis corresponds to cumulative exposure. The examples vary with respect to the age of migration (age of contact with L2, indicated by the dashed line) and the subsequent intensity of exposure to L1.

The final index of cumulative exposure reflected the time spent in Poland and in the United Kingdom in the lifetime of each child, as well as the amount of exposure the child received in each of these countries. The index of the cumulative exposure to Polish was calculated using the following formula: (time^4^ spent in Poland) ^∗^ 91 + (time spent in the United Kingdom) ^∗^ (exposure to Polish while in the United Kingdom). The actual unit of measurement used to calculate the index was the child’s age in days represented as years (in decimals). The mean cumulative exposure to Polish was 316.45 (*SD* = 93.64, range: 70.83–515.86). The index of cumulative exposure to English was calculated as: (the time spent in Poland) ^∗^ 0 + (the time spent in the United Kingdom) ^∗^ (the exposure to English while in the United Kingdom). The mean cumulative exposure to English was 158.85 (*SD* = 81.34, range: 16.87–362.13). **Figure 1** shows different possible scenarios of how language exposure can change with age influencing values of the cumulative exposure index.

The index of exposure will be used only in the regression analyses focusing on the bilingual group, which is the main focus of the present paper. We could not directly compare the monolingual and the bilingual groups with regards to exposure, because only parents of the bilingual children filled in the questionnaire concerning exposure to both languages.

### Statistical Analyses

As indicated earlier, in the analyses we focused on three central questions: (1) What are the differences between bilingual migrant children and their monolingual peers in the four domains of Polish L1 development? (2) How does the cumulative exposure to L1 and the cumulative exposure to L2 influence performance of the children in each language domain? (3) Can high exposure to L1 minimize the potential gap between monolinguals and bilinguals? To address the first question, we conducted a series of independent *t*-tests to compare the average scores of the bilingual and the one-to-one matched monolingual samples. To address the remaining questions, for each task we conducted a multiple regression analysis, exclusively on the bilingual sample. For the regression analyses we used the all-subsets method with *regsubsets()* function in the *leaps* package in R (Lumley and Miller, 2004) which performs an exhaustive search for the best regression model, containing a subset of predictors used in the maximal model. The maximal model contained cumulative exposure to Polish and the cumulative exposure to English as predictors, alongside with age, years of mother’s education, forward digit span, and Raven raw scores. The four latter factors were entered into the model to control for possible confound variables connected with cognitive development and SES. All the analyses were conducted on the subsamples of children to maximize the number of data points in the models – and thus the statistical power.

To test whether high exposure to Polish can minimize any performance gaps between monolinguals and bilinguals, for each task, we conducted additional analyses in which we selected a subset of 50% bilingual children with the highest weighted exposure^5^ to L1 (or the lowest exposure to L2, if L2 exposure was the significant factor) and compared them against their monolingual peers matched one-to-one (an analysis comparing the two groups on the full set of participants was not possible, see footnote 4). This regression analysis included two variables: Age and Group (monolingual, bilingual), and the interaction of Age and Group. A significant interaction would indicate that the magnitude of the gap between the groups changes with age.

**FIGURE 2:**
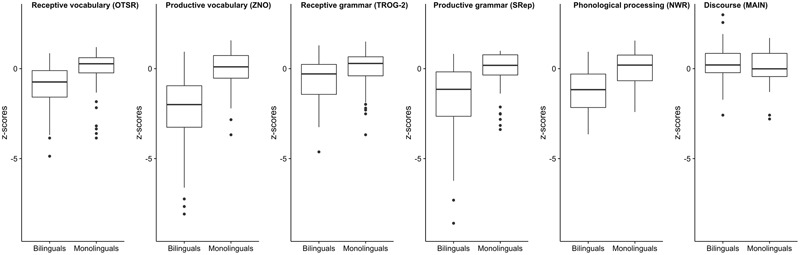
The average performance (in z-scores) of bilingual and monolingual groups in each language task. The z-scores were calculated on the basis of the mean and standard deviation of the monolingual group in each task.

**FIGURE 3:**
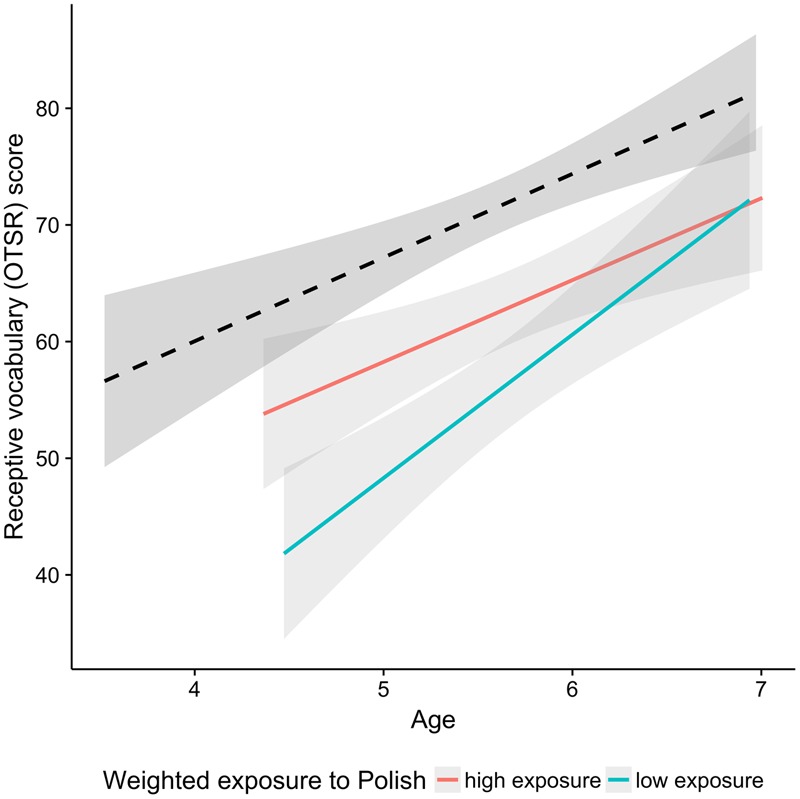
Scores in the receptive vocabulary test plotted as a function of age. The black dashed line indicates the monolingual group and the two colored lines correspond to the bilingual group. Red and aqua correspond to the median split on exposure to L1 Polish. The median split was performed for visualization purpose only.

**FIGURE 4:**
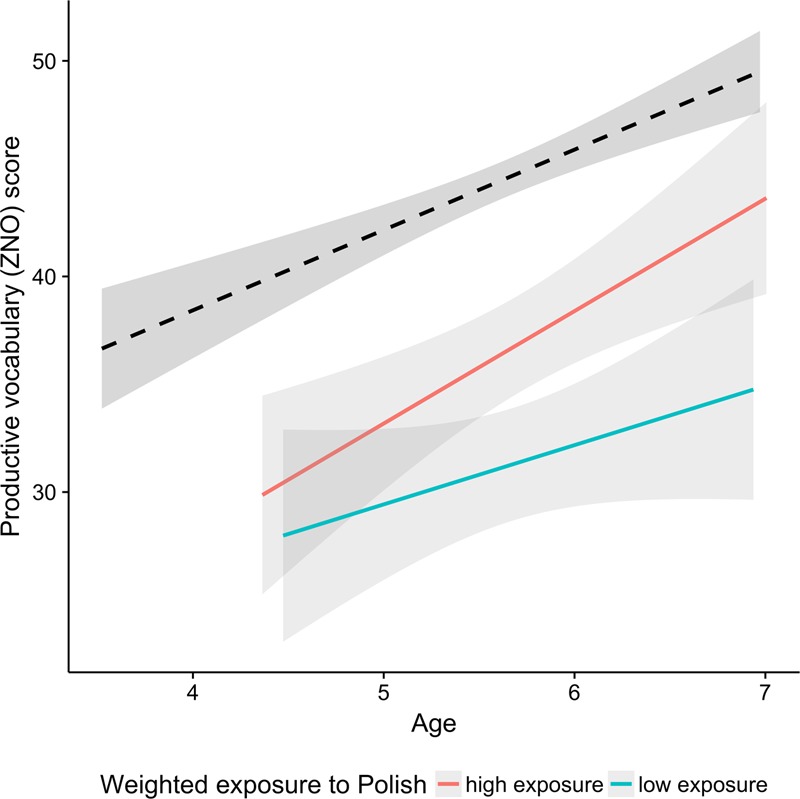
Scores in the productive vocabulary test plotted as a function of age. The black dashed line indicates the monolingual group and the two colored lines correspond to the bilingual group. Red and aqua correspond to the median split on exposure to L1 Polish. The median split was performed for visualization purpose only.

**FIGURE 5:**
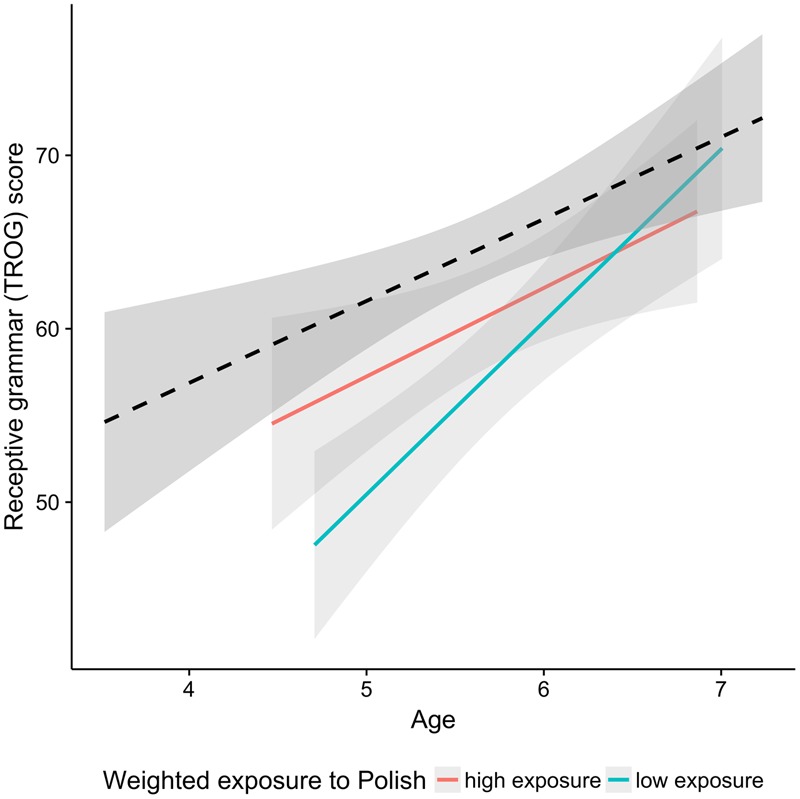
Scores in the receptive grammar test plotted as a function of age. The black dashed line indicates the monolingual group and the two colored lines correspond to the bilingual group. Red and aqua correspond to the median split on exposure to L1 Polish. The median split was performed for visualization purpose only.

**FIGURE 6:**
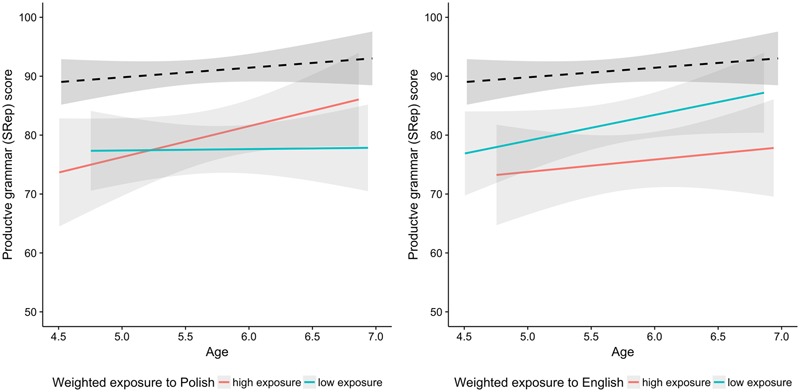
Scores in the productive grammar test plotted as a function of age. Black dashed line indicates the monolingual group and the two colored lines correspond to the bilingual group. The data for bilinguals is broken down by the median split amount of weighted exposure to Polish and English. Red and aqua correspond to the median split on exposure to L1 Polish (left side) and to L2 English (right side). The median split was performed for visualization purpose only.

To depict the effects of exposure and to visualize the comparison of performance between the monolingual and the bilingual group, for each task we overlaid the best-fit regression lines for the two groups, as a function of age (**Figures 2–6**). For the bilingual group, the regression line is broken down by a weighted estimate of exposure to Polish^5^ and this is consistently done for all graphs, regardless of whether cumulative language exposure to Polish turned out to be a significant predictor in the model. Additionally, whenever cumulative language exposure to English turned out to be a significant predictor in the model, we added a graph where the regression line is broken down by a weighted estimate of exposure to English.

## Results

**Figure 2** presents box plots showing the average performance of bilingual and monolingual groups in each language task. Although all the analyses were conducted on the raw scores, the graphs present the results converted to z-scores to allow easier comparison across different language measures. The z-scores were calculated on the basis of the mean and standard deviation of the monolingual group in each task.

### Receptive Vocabulary Test (OTSR)

On the receptive vocabulary task, the bilinguals scored on average 59.79 points out of 88 (*SD* = 14.03, range: 14–82), while the monolingual group scored on average 71.77 points (*SD* = 11.87, range: 26–86). The effect size as measured by Cohen’s d was large (*t*(172) = 5.99, *p* = 0.000, 95% CI [7.92, 15.69], *Cohen’s d* = 0.91).

**Table 2** presents the best regression model predicting the scores on the receptive vocabulary test in the bilingual group. The significant predictors in the model were Raven, digit span, and Polish cumulative exposure: the higher score in vocabulary test was related to higher IQ score, higher digit span, and greater cumulative exposure to Polish.

**Table 2:** The best regression model predicting the receptive vocabulary in the bilingual group.

	Estimate	*SE*	*t*	*P*
Intercept	15.04	5.59	2.69	0.009
Raven	0.96	0.22	4.38	0.000
Digit span	3.20	1.10	2.91	0.005
L1 (Polish) cumulative exposure	0.04	0.01	2.82	0.006

*F(3,83) = 22.92. *p* < 0.001, *Adj. R squared* = 0.43.*

**Figure 3** shows the difference in the receptive vocabulary scores depending on age, the amount of L1 exposure and group. A visual inspection of the figure suggests that a gap between the bilingual and monolingual children does not diminish with age, even in children with high exposure to Polish. A regression analysis with 50% of bilingual children with highest weighted exposure to Polish and their monolingual peers confirmed that the size of the gap between the monolingual and the high-exposure bilingual group does not diminish with age: There were significant main effects of Age and Group (*p* < 0.001), but no interaction (*p* > 0.3). The same type of regression analysis was repeated for other language tasks and is reported in the subsequent sections.

### Productive Vocabulary Task (ZNO)

On the productive vocabulary test, the bilingual group scored on average 34.13 points out of 53 (*SD* = 8.91, range: 6–49), while the monolinguals scored 44.52 points (*SD* = 4.77, range: 27–52). The difference between the groups was statistically large (*t*(172) = 9.59, *p* = 0.000, 95% CI [8.25, 12.53], *Cohen’s d* = 1.45).

**Table 3** presents the best regression model predicting the scores on the productive vocabulary task in the bilingual group. The significant predictors in the model were the Raven’s test scores and Polish cumulative exposure: the children with higher IQ, as well as those with higher cumulative exposure to Polish, had higher scores on the productive vocabulary test. **Figure 4** shows the increase in the scores with age for both monolinguals and bilinguals. Although the age-related increase in performance can be observed for children with both high and low levels of exposure to Polish, the children with high L1 exposure seem to benefit more. Still, there is a visible gap in performance between the monolinguals and bilinguals. A regression analysis with 50% of bilingual children with highest weighted exposure to Polish and their monolingual peers showed significant main effects of Age and Group (*p* < 0.001), but no interaction (*p* > 0.6). Therefore, while the gap between monolinguals and bilinguals seems smaller for the bilingual group with higher levels of exposure to Polish, the additional analyses do not provide any evidence that at high levels of L1 exposure, the gap can significantly decrease at later age.

**Table 3:** The best regression model predicting the productive vocabulary in the bilingual group.

	Estimate	*SE*	*t*	*P*
Intercept	12.49	3.52	3.48	0.001
Raven	0.42	0.14	2.88	0.005
L1 (Polish) cumulative exposure	0.04	0.01	4.43	0.000

*F(2,84) = 20.20, *p* < 0.001, *Adj. R squared* = 0.31.*

### Receptive Grammar Test (TROG-2)

On the receptive grammar task, the bilingual group scored on average 59.46 points out of 80 (*SD* = 10.86, range: 21–77), while the monolinguals scored 64.76 points (*SD* = 9.46, range: 30–79). The difference between the two groups was significant with a medium effect size (*t*(146) = 3.16, *p* = 0.002, 95% CI [1.99, 8.61], *Cohen’s d* = 0.52).

**Table 4** shows that the TROG scores were predicted by the Raven’s test scores and the digit span scores. Children who had higher scores on these tasks performed better on the receptive grammar test. Cumulative exposure to L1 (Polish) or L2 (English) was not included in the final model. As indicated by **Figure 5**, the gap in scores between the monolingual children and bilinguals is not very large and seems to decrease with age, particularly for the children with high exposure to Polish. An additional regression analysis conducted on 50% of bilingual children with highest weighted exposure and on matched monolingual peers revealed a main effect of Age (*p* < 0.01), but only a marginally significant effect of Group (*p* = 0.05), and no interaction of Group and Age (*p* = 0.89).

**Table 4:** The best regression model predicting the receptive grammar in the bilingual group.

	Estimate	*SE*	*t*	*P*
Intercept	27.47	4.36	6.30	0.000
Raven	0.83	0.18	4.55	0.000
Digit span	3.55	0.90	3.95	0.000

*F(2,71) = 28.32, *p* < 0.001, *Adj. R squared* = 0.43.*

### Productive Grammar Test (LITMUS-SRep)

When it comes to the productive grammar test, the bilingual group scored on average 76.12% out of 100 (*SD* = 17.48, range: 13.02–98.23), while the monolingual scores were close to ceiling (*M* = 90.80 point, *SD* = 9.05, range: 60.18–99.79). The effect size as measured by Cohen’s d was large (*t*(158) = 6.67, *p* = 0.000, 95% CI [10.33, 19.03], *Cohen’s d* = 1.05).

**Table 5** shows that the task results were predicted by the digit span, Raven scores, and L2 (English) cumulative exposure: the children with higher scores on STM and those with a higher IQ performed better on the SRep. However, the higher cumulative exposure to English resulted in the lower performance on the SRep test, as illustrated in **Figure 6**. There is a large gap in the performance on the task between the monolingual children and bilingual children with high exposure to English. The gap between the monolingual and bilingual children with low exposure to English is smaller. A regression analysis on 50% of bilingual children with the lowest weighted exposure to English and on matched monolingual peers revealed a significant effect of Age (*p* < 0.01) and of Group (*p* < 0.001), but the interaction between the two was non-significant (*p* > 0.7).

**Table 5:** The best regression model predicting the productive grammar in the bilingual group.

	Estimate	*SE*	*t*	*P*
Intercept	38.71	6.94	5.58	0.000
Raven	0.58	0.26	2.20	0.031
Digit span	9.11	1.46	6.24	0.000
L2 (English) cumulative exposure	-0.08	0.02	-4.36	0.000

*F(3,76) = 25.75, *p* < 0.001, *Adj. R squared* = 0.48.*

### Phonological Processing Task (NWR)

On the NWR task, the bilingual group scored on average 22.51 points out of 50 (*SD* = 9.23, range: 3–40) and the monolinguals scored 32.41 (*SD* = 8.06, range: 13–45). The effect size, as measured by Cohen’s d, was large (*t*(156) = 7.18, *p* = 0.000, 95% CI [7.17, 12.62], *Cohen’s d* = 1.14).

**FIGURE 7:**
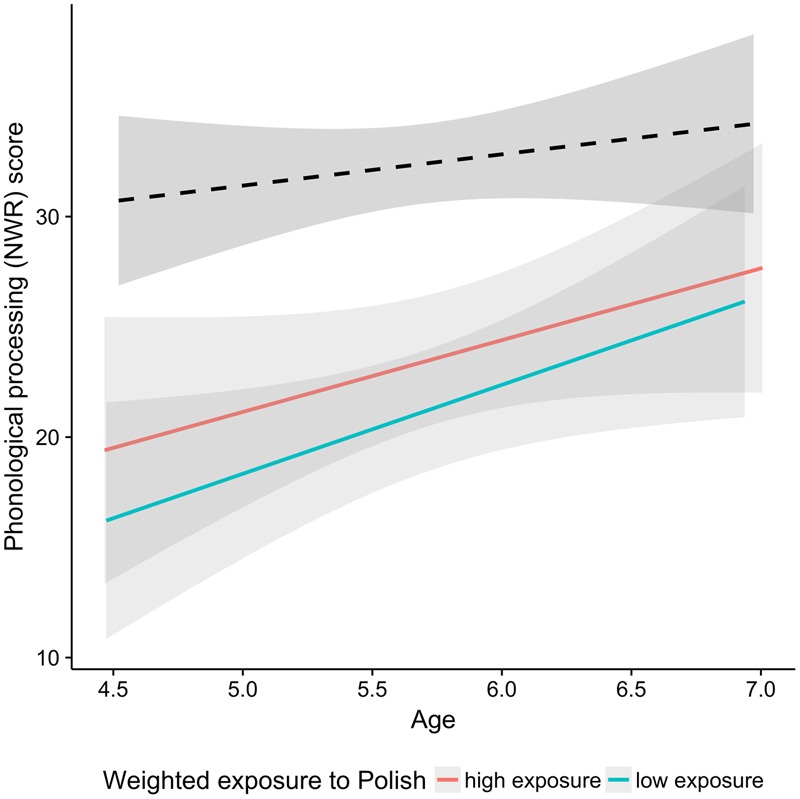
Scores in the phonological processing test plotted as a function of age. The black dashed line indicates the monolingual group and the two colored lines correspond to the bilingual group. Red and aqua correspond to the median split on exposure to L1 Polish. The median split was performed for visualization purpose only.

**Table 6** shows that children with the higher digit span score and those with higher cumulative exposure to Polish had higher NWR scores. As indicated by **Figure 7**, the gap between the bilingual and monolingual children is lower for the bilinguals who had higher exposure to Polish. However, even for those children the gap does not seem to disappear with age, as also indicated by a regression analysis with 50% of bilinguals with highest weighted exposure to Polish and their matched monolingual peers. While there was a significant effect of Age (*p* < 0.01) and Group (*p* < 0.001), there was no significant interaction between them (*p* > 0.69).

**Table 6:** The best regression model predicting the phonological processing in the bilingual group.

	Estimate	*SE*	*t*	*P*
Intercept	-2.88	4.76	-0.60	0.547
Digit span	4.53	0.92	4.90	0.000
L1 (Polish) cumulative exposure	0.02	0.01	2.29	0.024

*F(2,76) = 15.56, *p* < 0.001, *Adj. R squared* = 0.27.*

### Discourse Task (LITMUS-MAIN)

In terms of the MAIN task, the bilingual group scored on average 8.13 points out of 17 for the story structure in the Telling condition (*SD* = 2.86, range: 1–16) and 9.21 points for the story structure in the Retelling condition (*SD* = 3.18, range: 3–17). The monolingual group scored on average 7.36 points for the story structure in the Telling condition (*SD* = 2.71, range: 3–13) and 8.68 points for the story structure in the Retelling condition (*SD* = 2.98, range: 0–14). The Telling and Retelling scores correlated moderately in both groups (bilinguals: *r* = 0.45, *p* = 0.001, monolinguals: *r* = 0.34, *p* = 0.01), therefore, for the further analyses we averaged the scores from the Telling and Retelling part of the task. When the scores were averaged, the bilingual group scored on average 8.63 points (*SD* = 2.57, range 2–15), while the monolinguals scored 8.02 points (*SD* = 2.33, range 1.5–12). The difference was not statistically significant, and the effect size as measured by Cohen’s d was negligible (*t*(104) = 1.37, *p* = 0.175, 95% CI [-0.29, 1.60], *Cohen’s d* = 0.27).

**FIGURE 8:**
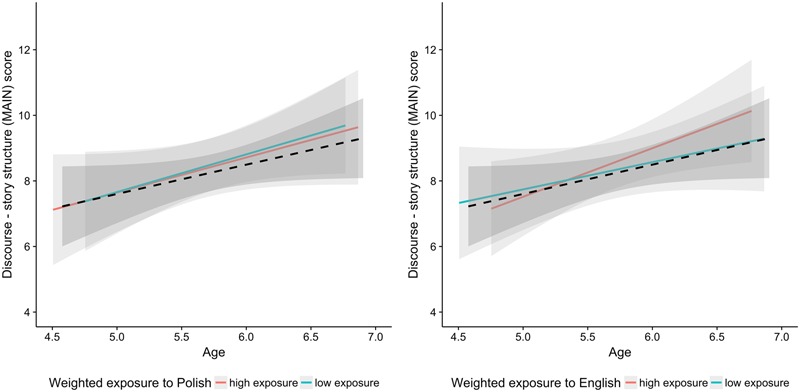
Scores on the story structure plotted as a function of age. Black dashed line indicates the monolingual group and the two colored lines correspond to the bilingual group. The data for bilinguals is broken down by the median split amount of weighted exposure to Polish and English. Red and aqua correspond to the median split on exposure to L1 Polish (left side) and to L2 English (right side). The median split was performed for visualization purpose only.

**Table 7** shows that the children with higher cumulative exposure to Polish and the children with higher cumulative exposure to English constructed more well-formed stories. This result is illustrated in **Figure 8**. The bilingual children with low exposure to Polish perform similarly to monolingual children on the task. The bilingual children with high exposure to Polish seem to score even higher than monolinguals.

**Table 7:** The best regression model predicting performance in the discourse task (story structure) in the bilingual group.

	Estimate	*SE*	*t*	*P*
Intercept	0.45	2.17	0.21	0.836
L1 (Polish) cumulative exposure	0.02	0.00	4.01	0.000
L2 (English) cumulative exposure	0.01	0.01	2.38	0.021

*F(2,50) = 8.46, *p* < 0.001, *Adj. R squared* = 0.22.*

## Discussion

In this paper, we examined language skills in L1 Polish of Polish-English bilingual children (aged 4–7 years) growing up in the United Kingdom. We focused on four language domains: vocabulary (receptive and productive), grammar (receptive and productive), phonological processing, and discourse production (narration). We compared the overall scores in each task between bilinguals and monolinguals matched one-to-one on age, gender, maternal education, non-verbal IQ, and STM span. Further, in a series of regression analyses, we investigated the effect of cumulative exposure in L1 and L2 on the task scores, controlling for general cognitive abilities (non-verbal IQ and STM span), as well as SES and age. Finally, with another set of regression analyses we explored whether a greater amount of L1 exposure could possibly diminish the gap between the bilingual and monolingual children. Below, we first consider the results with regard to the overall performance of the bilinguals and monolinguals, and then focus on the contribution of language exposure to the language outcomes in the bilingual group.

### Differences between Bilingual Migrant Children and Their Monolingual Peers in the Four Domains of L1 Polish

The overall finding of our study is that in their performance on most L1 measures the bilinguals lagged behind their monolingual peers. There were large differences between the groups in terms of productive vocabulary, productive grammar (as measured by the SRep task), as well as phonological processing (as measured by the NWR task). There were also moderate differences between the groups in terms of receptive vocabulary and receptive grammar. However, the bilingual group did not differ from monolinguals in terms of story structure coherence in the narrative task. The results obtained are, to a large extent, consistent with the previous findings on L2 development in bilingual children.

With respect to the vocabulary size, previous research indicates that when tested in one language, bilingual children have smaller productive and receptive vocabulary than monolinguals (Pearson et al., 1993; O’Toole et al., 2017), even when tested in their L1 (e.g., Pearson et al., 1997; Uccelli and Páez, 2007; Miękisz et al., 2017). Our study adds new evidence to this body of research. It also provides new insights into identifying the sources of performance gap between bilinguals and monolinguals thanks to including a carefully matched monolingual control group. We have demonstrated that bilingual children have a smaller vocabulary in L1 than their monolingual peers even when their SES and general cognitive abilities are comparable.

The bilinguals also scored lower than monolinguals on both receptive and productive grammar tasks. This result replicates previous findings, showing developmental difficulties in L1 grammar among bilingual children (Thordardottir and Brandeker, 2013). We also observed that for the bilinguals (but not for the monolinguals) the productive grammar task was more difficult than the receptive grammar task. This result reflects the pattern that has been reported previously in studies on L2 grammar performance in bilinguals. It shows that children struggle with the production of grammar, even if they have the receptive knowledge of the grammatical constructions tested (Chondrogianni and Marinis, 2012).

The large gap between the bilingual and monolingual children on the NWR task was more surprising, since many previous studies reported children scoring better on this phonological processing measure in L1 than in L2 (Gutiérrez-Clellen and Simon-Cereijido, 2010; Summers et al., 2010). However, the NWR task used in our study might be more sensitive to problems with L1 phonological processing, since it deliberately contained many phonological structures typical for Polish. This might have resulted in the effect obtained for bilinguals on NWR, in contrast to previous research, which utilized various types of quasi-universal tasks. Delays in L1 phonological development among bilingual children have been reported before, which makes this explanation plausible (Goldstein and Washington, 2001; Fabiano-Smith and Barlow, 2010).

As far as the discursive abilities are concerned, in the MAIN task, the bilinguals scored on par with their monolingual peers for the story structure of their narratives, which replicated the finding by Kunnari et al. (2016). This result can be explained by the fact that the narrative abilities intersect children’s language abilities and their pragmatic awareness (Reese et al., 2010). Telling a coherent narrative requires robust cognitive skills necessary for building a logical storyline, so children’s discourse abilities probably go beyond their language-specific skills (Paradis et al., 2014; Gagarina et al., 2016). Previous studies have shown that similar age-dependent narrative patterns are shared by monolingual children from different language backgrounds (Berman and Slobin, 1994) and it seems that narrative abilities develop similarly in bilingual and monolingual children.

### The Impact of the Cumulative Exposure to L1 and L2 on Language Performance across the Four Domains

The second set of findings relates to the effects of exposure on language measures. We have found that the cumulative exposure to L1 was related to higher scores in the receptive vocabulary, productive vocabulary, phonological processing, and discourse. We also found an adverse effect of L2 cumulative exposure on only one language measure – the productive grammar, and its seemingly surprising positive effect on the narrative production. For the receptive grammar, we found no significant effect of exposure to L1 or to L2 once other factors have been controlled for.

Overall, the results suggest that language exposure is crucial primarily for the productive tasks (producing grammar and vocabulary, repeating non-words and producing narratives) and has less of an impact on the comprehension tasks. This finding is in line with the previous research on bilingual children that shows the influence of language exposure on productive tasks in L1 (Thordardottir and Brandeker, 2013 – SRep; Patterson, 2002 – vocabulary; Summers et al., 2010 – NWR). Moreover, it aligns with an earlier study by Thordardottir (2011), who found that although language exposure influenced both the receptive and productive vocabulary scores of bilingual children, the effect was much greater for the productive tasks. While the complete lack of effect of exposure on the receptive grammar tasks contradicts previous research (Gathercole and Thomas, 2009; Thordardottir, 2011), this discrepancy might be due to the fact that previous studies did not fully control for the factors related to the cognitive development of children, such as the non-verbal IQ and STM span. In our study, the two factors strongly predicted the receptive grammar scores, and these general cognitive abilities explained most of the variance in this language task.

The differential effects of the cumulative exposure on the receptive and productive tasks found in the current analysis are of vital importance, because they suggest that exposure to the home language is critical for mastering the productive skills in this language. It also appears that the performance in the receptive tasks is much less impacted by the amount of language exposure: it is easier to understand than to produce language having had little exposure to that language. It is also worth adding that the inter-subject variability in the receptive grammar performance was much smaller than in the production task and so was the performance gap between monolinguals and bilinguals.

Another issue is the negative effect of L2 exposure on the L1 production of grammatical structures in the SRep task. This result suggests the existence of negative transfer from L2 to the L1 (Paradis and Navarro, 2003; Bernardini and Schlyter, 2004; Kupisch, 2007). More specifically, when repeating Polish sentences in the SRep task, which involves accessing the mental representation of a given structure, the knowledge of English syntactic templates possibly interfered with the knowledge of Polish syntax, leading to errors in the production of syntactically complex Polish sentences. Another possible explanation is that the early acquisition of English, a language less morphosyntactically complex than Polish, “desensitized” children to the complexity of Polish inflection (van der Slik et al., 2017). However, at this point the above interpretations of the negative effect of L2 exposure on the scores in L1 SRep task are only speculative. A qualitative error analysis would be required to determine the precise sources of difficulty in SRep. Thus, further research is needed to determine in what ways L2 exposure may affect L1 grammatical performance.

A separate question is why there was no impact of exposure to L1 Polish on the performance in the SRep task. One hypothetical explanation is purely statistical: if the indices of exposure to L1 and to L2 were highly collinear, introducing one of the indices might have “pushed out” the other from the model. However, in this case the two indices share little common variance (14%), so this explanation is rather unlikely. It is thus more plausible that the L1 input typically directed to bilingual children in the migrant context does not systematically familiarize them with the syntactic knowledge required to repeat more complex sentences (e.g., object and subject relative clauses, conditionals, object and subject clefts and noun complement clauses). Hence, the large variability in the SRep scores in the bilingual children and the absence of any impact of L1 exposure. In contrast, the monolingual children might systematically be exposed to such structures not only at home, but also in educational settings and through the media, which would explain why their scores were higher and less varied. However, more research is needed on the features of home discourse and its relationship with children’s syntactic development to further substantiate this claim.

At the same time, there is a positive influence of L2 exposure on the discourse production, as the bilingual children’s narrative abilities are positively correlated with both L1 and L2 exposure. This is consistent with the previous research, which suggests that the ability to create coherent stories is independent of the language-specific skills (e.g., Iluz-Cohen and Walters, 2012; Gagarina, 2016; Rodina, 2016). If the ability to tell coherent stories is less reliant on the specific language skills, but depends on the child’s pragmatic awareness (Reese et al., 2010), such an awareness develops in contact with any of the two languages of the bilingual child. Possibly, in the initial years of schooling, there is a carry-over of the child’s narrative abilities across the two languages, even if the child’s linguistic abilities in one of the languages are weaker (Gagarina, 2016). This suggests that in child bilinguals, exposure to any language builds a language-universal ability to structure stories in a coherent way.

### Can High Exposure to L1 Minimize the Potential Gap between Monolinguals and Bilinguals?

While the high exposure to L1 positively influenced language outcomes in the bilingual children, our study suggests that it might not be enough to minimize the gap between the monolingual and bilingual children. For each task in which we observed the effect of cumulative exposure to L1, we conducted an additional analysis on the bilingual children with the highest weighted L1 exposure and a matched monolingual group. In the analyses, we tested for an interaction between group and age on the task outcomes. The presence of such an interaction would indicate that with age, the bilingual children with high rates of L1 exposure “catch up” with their monolingual peers. Contrary to our expectations, we have not observed such an effect for any of the tasks where a performance gap was observed. It is interesting to note that this result was consistent across the board – i.e., for grammatical, lexical and phonological tasks, both productive and receptive. This indicates that even though exposure might be more crucial for productive rather than receptive tasks, there is no domain in which high exposure to L1 guarantees outcomes comparable to that of monolingual peers. Overall, the results suggest that although exposing bilingual children to L1 will certainly benefit their L1 language performance, it might not be enough to minimize gaps in L1 skills between them and their monolingual peers.

## Conclusion

Our study shows that when tested in L1, bilingual children lag behind their monolingual peers on vocabulary, grammar, and phonological processing. The performance differences between the two groups are most prominent in the productive language tasks. At the same time, the productive tasks are also more influenced by cumulative language exposure than the receptive tasks. While high exposure to L1 might not be enough to close the performance gap between the bilingual and monolingual children, providing exposure to L1 and promoting situations in which bilinguals could practice their production in L1 will certainly benefit their development in that language. This finding is essential not only for furthering our understanding of bilingual language acquisition, but it also has important practical implications. Unlike many other migrant communities who stay in the host country for good, Polish families often re-migrate to Poland, the home country of the parents. Upon returning to their home country, many children of Polish migrants experience educational setbacks due to inferior knowledge of their L1 Polish, as compared to their monolingual peers (Grzymała-Moszczyńska et al., 2015). Our study points to the areas where these children might experience most difficulties – namely productive vocabulary and grammar. It also shows that extensive and varied exposure to L1 in these areas would certainly be beneficial. These clues might be used to design better interventions for the migrant children who return to their home countries and who face language difficulties in their L1. It also shows that narration (story structure) is a strength in bilinguals’ L1 performance. Hence the interventions could build on this strength when trying to enhance vocabulary and grammar.

The results of the current study provide support for the claims made in the heritage language literature that the L1 of young heritage language speakers resembles more an L2 learned in the adulthood, than an L1 naturally acquired in the childhood (e.g., Rothman, 2009). Heritage speakers usually end up being dominant in the majority language in adulthood, no matter whether both their languages are present from birth, or the majority language is acquired later due to migration in childhood (Cabo and Rothman, 2012). The results reported in the current study suggest that the Polish-English bilinguals growing up in the United Kingdom may never reach the level of a monolingual Polish peers growing up in Poland. Such “incomplete L1 acquisition” is defined by Montrul (2008, p. 21), as “a mature linguistic state, the outcome of language acquisition that is not complete (...) in childhood (...), when some specific properties of the language do not have a chance to reach age-appropriate levels of proficiency after intense exposure to the L2 begins.” We speculate that such a scenario is likely, if the Polish-English bilinguals stay in the United Kingdom for good and maintain only sporadic contacts with their Polish-speaking families in Poland.

Our study is not without limitations. First, our bilingual sample is not fully representative of the population of Polish migrant children in the United Kingdom. The families who took part in our research were volunteers, which means they were possibly interested in the subject of bilingualism (Haman et al., 2014). Thus, our data set did not include a large part of the Polish migrant population in the United Kingdom who do not support maintaining L1 in their children (e.g., for the sake of acculturation and integration with the ambient society). It is therefore likely that our data paint an overly optimistic picture of the L1 performance in bilingual child migrants. The second limitation is that the current report includes only the analyses of L1 performance, but no analogous analyses L2 performance, which will be completed in the near future. The last limitation is that both languages of the bilingual children are Indo-European and both follow the canonical SVO word order. It is thus not clear how our findings can translate to pairs of more typologically distinct languages.

Nevertheless, the reported study is unique in that it presents a comprehensive analysis of bilingual children’s L1 across a range of language domains. An additional value of the study is that the performance of the bilingual migrant children was compared with that of carefully matched monolinguals. We were also able to isolate the impact of language exposure in both L1 and L2 on language skills, while at the same time controlling for a range of other factors known to contribute to performance in language tasks. In the future, we plan to extend our exploration by conducting more detailed error analyses on the collected language material. This should reveal the most problematic areas which account for the gap between monolinguals and bilinguals in the domains of vocabulary, grammar, and phonological processing. In particular, the analysis of errors in sentence comprehension and production should be valuable as it will shed more light on the issue of cross-linguistic influence between the two languages of a bilingual.

## Ethics Statement

Study presented in this paper was approved by the Komisja ds. Etyki Badań Naukowych (Ethics Committee) at the Faculty of Psychology, University of Warsaw. Parents of all children who participated in the study signed informed consent forms. Children gave oral assent to take part in the study.

## Author Contributions

Leading the main project: EH and ZW (both first authors). Leading the supporting projects: ZW and AO. Conception and design of the study: EH, ZW, JS, MB-P, AO, and AM. Design of tasks used in the study: EH, ZW, JS, MB-P, AO, and NB. Data collection: KM, JK, AK, AM, MF-N. Data coding and analysis: EH, ZW, MM, JS, MB-P, AO, KM, MŁ, JK, AM, AK, NB, and MF-N. Data interpretation: EH, ZW, MM, JS, MB-P, AO, KM, MŁ, JK, AM, AK, NB, and MF-N. Drafting the article: MM and JS. Critical revision and rewriting of the article: EH, ZW, MM, JS, MB-P, AO, KM, MŁ, JK, AM, AK, NB, and MF-N. Final approval of the version to be published: EH, ZW, MM, JS, MB-P, AO, KM, MŁ, JK, AM, AK, NB, and MF-N.

## Conflict of Interest Statement

The authors declare that the research was conducted in the absence of any commercial or financial relationships that could be construed as a potential conflict of interest. The reviewer, MR declared a past co-authorship with two of the authors, MŁ and EH to the handling Editor.

## Footnotes

1 Throughout the paper, we use the term, “majority language” or L2 for the language of the country where the bilingual children of the migrants live (in the case of this study – English). We use the term “minority language,” “home language” or “L1” for the language used by at least one the child’s parents (in the case of this study – Polish).

2 We slightly modified the original instruction to make it friendlier for children younger than 6-year-olds.

3 The issues concerning the exposure to English and to Polish were not interdependent. More specifically, the parents could indicate that the child had a large exposure to both L1 and L2, or that the child had little exposure to both languages. In consequence, the estimates of exposure to L1 and L2 were only moderately correlated (*r* = -0.56, *p* < 0.001).

4 Our measure took into the account not only the years, but also the months and the days. The months and the days were represented in decimal values. For instance, a child could spend 2.42 years in the United Kingdom (i.e., 2 years, 5 months, and 3 days).

5 The weighted estimate of exposure is simply the cumulative exposure in a given language divided by age. We use this index (rather than the cumulative exposure index) as a base of the median split for the purpose of visualization, because the graphs are plotting the data already as a function of age.
